# Bilateral torsion of fallopian tubes with bilateral hydrosalpinx: a case report

**DOI:** 10.1186/s13256-020-02445-2

**Published:** 2020-08-05

**Authors:** Taner Kartal, Ozer Birge

**Affiliations:** 1 Department of Gynaecology and Obstetrics, Urfa State Hospital, Urfa, Turkey; 2 Department of Gynaecology and Obstetrics, Nyala Sudan Turkey Training and Research Hospital, Nyala - Darfur, Sudan

**Keywords:** Fallopian tube, Bilateral, Torsion, Acute pelvic pain

## Abstract

**Background:**

Isolated fallopian tube torsion is a very rare cause of acute abdominal pain in women and, as can be expected, its being bilateral is an extremely rare condition. It is more common in women in reproductive age compared to other age groups. Symptoms, physical examination, imaging and laboratory findings being nonspecific makes it difficult to establish the correct diagnosis and often the diagnosis can be made during surgery. Despite being a very rare condition in general, it is important in terms of preservation of tube and thus the fertility especially in women of reproductive age with early diagnosis and treatment. Therefore, keeping in mind the fallopian tube torsion among the differential diagnoses in women presenting with acute abdominal pain will contribute to early diagnosis and treatment.

**Case presentation:**

A 38-year-old white Arabian woman, gravida 1, parity 0, abort 1, sought medical advice in our outpatient clinic with a complaint of lower abdominal pain that had started 2 days earlier. The pain had first started as mild cramps, which then suddenly intensified nearly 2 hours before her presentation to our clinic, spread to the groin and femur, more prominent on the right side, and became an ongoing pain. As preoperative diagnoses of the patient, ovarian cyst rupture and ectopic pregnancy were suspected, and fallopian tube torsion was also suspected due to the normal appearance of the ovaries and the appearance of the hydrosalpinx on ultrasonography. The patient underwent laparotomy with a Pfannenstiel incision. Both tubes had hydrosalpinx, and the fimbrial ends were blunt and obliterated. Bilateral salpingectomy was performed because the right tube had a prominent necrotic appearance, and there was a significant hydrosalpinx in both tubes.

**Conclusion:**

Bilateral fallopian tube torsion should be considered among the differential diagnoses in women presenting with acute pelvic pain.

## Background

Isolated fallopian tube torsion occurs when the fallopian tube revolves around its longitudinal axis without affecting the ovarian blood and lymphatic stream. This is a very rare condition among the causes of acute abdominal pain in women. Its incidence has been reported as 1 in 1,500,000 [1, 2]. It was first described by Bland-Sutton in 1890 [3, 4].

Despite being a very rare condition, isolated fallopian tube torsion is important in terms of creating acute abdominal pain, with surgery being the definitive method of treatment and preservation of the tube, and thus fertility, with early diagnosis and treatment being important especially in women of reproductive age. Tubal torsion is more commonly seen on the right side. This is probably due to the fact that the mobility of the left tube is partly less than that of the right side due to its proximity to the sigmoid mesentery, and right lower quadrant pain is evaluated more frequently with surgical exploration due to suspicion of appendicitis [4]. When considering the prevalence of unilateral fallopian tube torsion, it is easily understood that bilateral torsion of the fallopian tubes is an extremely rare condition. A review of the literature revealed that bilateral fallopian tube torsions are seen very rarely in case reports, and some of these cases are asynchronous torsions of both tubes [5–8]. In this case report, we describe a patient with bilateral fallopian tube torsion and bilateral hydrosalpinx who presented to our clinic with severe lower abdominal pain and was surgically treated, and we present a review of the literature.

## Case presentation

**Fig. 1:**
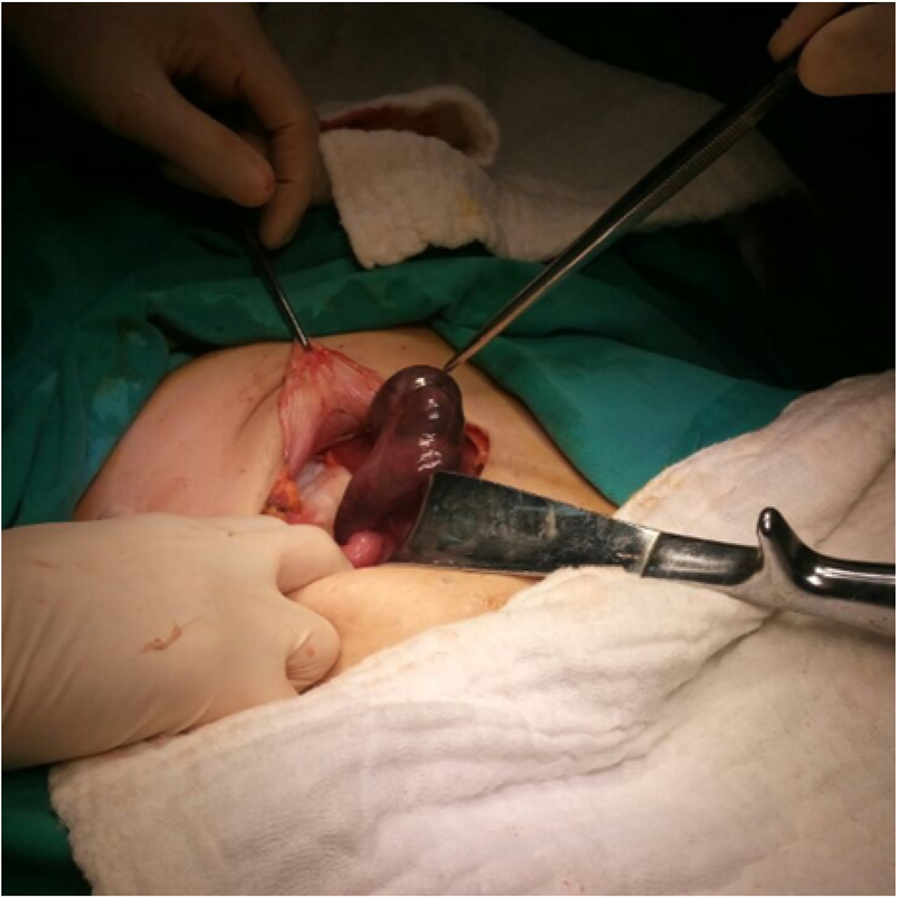
Right tube with necrotic appearance

**Fig. 2:**
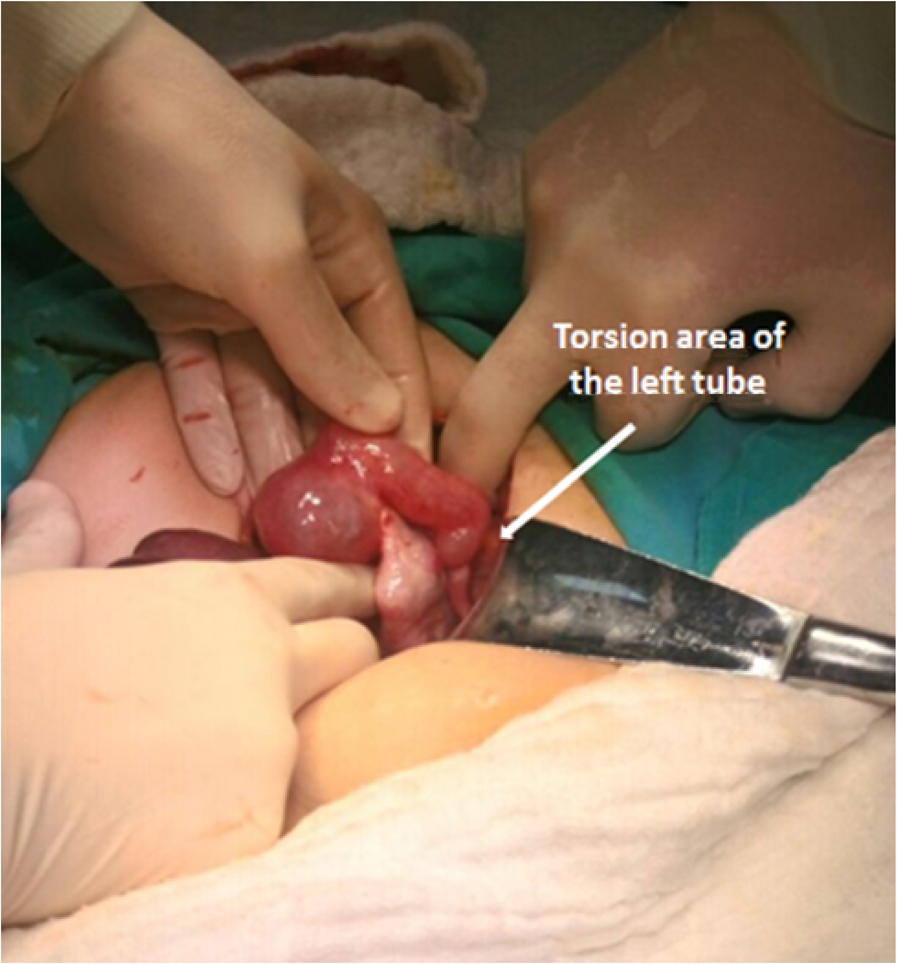
Left tube and torsion area and normal-looking left ovary

A 38-year-old white Arabian woman, gravida 1, parity 0, abort 1, sought medical advice in our outpatient clinic with complaints of lower abdominal pain that had started 2 days earlier. The pain had first started as mild cramps, which then suddenly intensified nearly 2 hours before her presentation to our clinic, spread to her groin and femur, more prominent on the right side, and became an ongoing pain. The patient had not had any complaints of pain until 2 days earlier in her anamnesis, and she stated that her nausea had started together with the intensification of pain, but she had not vomited. In her medical history, the patient stated that a doctor she had sought medical advice from for infertility about 3 years ago had recommended bilateral salpingectomy and *in vitro* fertilization treatment due to hydrosalpinx, but the patient did not undergo these procedures. She had undergone no previous abdominal operation. In the patient’s physical evaluation, we measured TA 110/70 mmHg, pulse rate 95 beats/minute, and no fever. The patient’s abdominal examination revealed significant defenses and rebounds in the lower abdominal quadrants, and significant sensitivity was detected in the upper and middle quadrants. Cervical movements were painful during the gynecologic examination. Transvaginal and abdominal ultrasonographic (US) examinations revealed that the uterus was normal, both ovaries were separated and normal, and two irregular cystic masses of 35 mm and 40 mm (possibly hydrosalpinx) were observed in the region close to the left adnexal lobe, and minimal free fluid was seen in the pouch of Douglas. Laboratory test findings were as follows: white blood cell count 9.46 × 10^3^/mm^3^, hemoglobin 9.3 g/dl, hematocrit 28.67%, and β-human chorionic gonadotropin (β-hCG) detected as negative. Tumor marker values were within normal limits (CA 125, 8.6 U/ml; CA 15-3, 12.08 U/ml; CA 19-9, 9.73 U/ml; carcinoembryonic antigen, 1.57 ng/ml). A decision was made to perform emergency surgery because acute abdominal findings were apparent, with the patient describing severe pain, clinical findings progressing, and adnexal pathology being detected by US. As preoperative diagnoses of the patient, ovarian cyst rupture and ectopic pregnancy were suspected, and fallopian tube torsion was also suspected due to the normal appearance of the ovaries and the appearance of hydrosalpinx by US. The diagnosis of ectopic pregnancy was excluded because of the negative β-hCG finding. The patient underwent laparotomy with a Pfannenstiel incision. The uterus and both ovaries were normal in abdominal observation. Hydrosalpinx was found in the right tube, and it was torsioned around itself four times and necrotic (Fig. 1). In the left tube, hydrosalpinx and 1.5 times torsion around itself were observed. The left tube was torsioned exactly at the junction of the distal hydrosalpinx and the normal proximal tubal region. There was no apparent necrotic appearance in the left tube, possibly due to the fact that circulatory disruption was not complete (Fig. 2). Both tubes had hydrosalpinx, and the fimbrial ends were blunt and obliterated. Bilateral salpingectomy was performed on the patient because the right tube had a prominent necrotic appearance, and there was a significant hydrosalpinx in both tubes. The patient was discharged on the second postoperative day without any complaints and without any complications. Histopathological examination revealed bilateral hydrosalpinx with hemorrhagic infarction findings consistent with torsion.

## Discussion

Isolated fallopian tube torsion is a rare cause of acute abdominal pain in women. It is primarily seen in adolescent and reproductive age women and is rarely encountered in the postmenopausal period. It is also seen less frequently in the pediatric age group than in women in the reproductive period [1, 4]. Isolated fallopian tube torsion is more common in women of reproductive age than in other age groups, probably because risk factors for tubal torsion, such as ovarian cysts, infections, and pelvic surgery, occur more frequently in women in the reproductive age group [9]. Although the etiology is not known for certain, the etiologic factors, which were divided into two groups as intrinsic and extrinsic factors, have been asserted. Intrinsic causes are factors intrinsic to tubes that contain congenital tubal abnormalities, hydrosalpinx, hematosalpinx, tubal neoplasms, and primary tubal surgeries such as tubal ligation. Ovarian and paratubal masses, pregnancy, trauma, adhesions, and pelvic congestion are reported as extrinsic factors [10]. One of the intrinsic factors, bilateral hydrosalpinx, was present in our patient.

Clinical signs of tubal torsion include lower abdominal pain, nausea, vomiting, urinary complaints, susceptible adnexal mass, and uterine bleeding. The most common symptom is pain that begins in the lower abdomen or pelvis on the affected side and may also spread to the back, thigh, or groin areas. The properties of the pain may be continuous and ambiguous, as well as paroxysmal and knifelike. In addition, defense and rebound can be detected on the torsion side. However, none of these are specific properties [4, 9, 11, 12]. Preoperative diagnosis of isolated fallopian tube torsion is difficult due to symptoms and physical examination findings not being pathognomonic and lack of specific imaging and laboratory features. Therefore, the correct diagnosis is often made during surgical intervention. Regarding the difficulty in making a preoperative diagnosis, Lo *et al.* reported that none of 17 patients with isolated fallopian tube torsion were diagnosed preoperatively [9, 12–14]. Because most patients with isolated fallopian tube torsion describe lower abdominal and lateral pain, the differential diagnosis should include acute appendicitis, ovarian cyst rupture or torsion, ectopic pregnancy, pelvic inflammatory disease, endometriosis, leiomyoma degeneration, intestinal obstruction or perforation, and renal colic [11, 13]. Although fallopian tube torsion is mostly symptomatic, cases that are asymptomatic have also been reported in the literature. For example, Murphy *et al.* reported a case of fallopian tube torsion detected incidentally during laparoscopy [15]. In this regard, they stated that spontaneous bilateral fallopian tube torsion may appear as primary infertility without any symptoms beforehand; therefore, it should be considered in the differential diagnosis of patients with bilateral tubal obstruction or bilateral hydrosalpinx [15]. Although fallopian tube torsion is more common in women in the reproductive period, it should not be forgotten that it may occur in pediatric patients, though rarely. It can often be misdiagnosed at a pediatric age. As an extremely rare case, Lima *et al.* reported that they diagnosed bilateral hydrosalpinx and asynchronous fallopian tube torsion in a 13-year-old premenarchal girl with lower quadrant pain [16].

The first imaging method used in most of the women with acute pelvic pain is US because of the lack of radiation exposure as well as its cost-effectiveness and noninvasiveness. Although US features may vary in patients with tubal torsion, detection of a tapering, elongated, and curled cystic mass as it comes close to the uterine horn may suggest the diagnosis of tubal torsion. Doppler sonography may also be helpful in the differential diagnosis. Although normal vascular flow is observed in the ovaries, a lack of diastolic flow or an observation of diastolic reverse stream together with high-impedance arterial stream in Doppler US of the adnexal mass wall may increase the suspicion of tubal torsion. However, the observation of a normal stream form by Doppler US does not always exclude the torsion [1, 11, 17, 18]. In our patient’s case, tubal pathology was suspected because of the normal appearance of the ovaries separately and detection of an irregular cystic mass in the adnexal region by US, and tubal torsion was also considered among the preoperative differential diagnoses. Doppler US was not performed in our patient’s case; in addition, no imaging method other than US was performed. Although imaging methods are generally helpful in patients with acute abdominal pain, fallopian tube torsion is rarely diagnosed preoperatively [13]. However, Fadıloğlu *et al.* reported that five patients with lower abdominal pain of different severities were diagnosed with preoperative tubal torsion using US alone; no other imaging method other than US was used, and these diagnoses were confirmed by laparoscopy [17]. In our opinion, especially in women with acute abdominal pain who are of reproductive age, tubal torsion can be seriously suspected by physical examination and US evaluation if considered among the differential diagnoses.

The treatment of fallopian tube torsion is surgery. Because most of the patients are young and in the reproductive period, if the torsioned tube is not necrotic and there is no evidence to suggest malignancy, tubal detorsion should be applied as a fertility-protecting surgery. However, if the fallopian tube appears to be necrotic, an adnexal neoplasm is detected, or the patient has completed fertility, salpingectomy may be performed. In isolated tubal torsion, difficulty in excluding differential diagnoses may lead to delayed surgical intervention. Therefore, fallopian tube necrosis is frequently encountered, and salpingectomy may be required for treatment. However, if surgery is performed without delay, fallopian tube protective surgery can be applied [9, 13]. Bilateral salpingectomy was performed in our patient because of the necrotic appearance of the right tube, the presence of hydrosalpinx in both tubes, and nonfunctional appearance.

## Conclusion

Fallopian tube torsion should be considered among the differential diagnoses in women presenting with acute pelvic pain. Although it is a rare condition, it should be kept in mind especially in adolescent and reproductive age women, and it is important in terms of early surgical treatment and protection of fertility.

## Data Availability

The authors agree to make the raw data and materials described in this report freely available.
